# Supplementary material for: Metabolite profiles associated with disease progression in influenza infection
Source: PLoS One. 2021 Apr 2;16(4):e0247493. doi: 10.1371/journal.pone.0247493 (PMC8018623; doi:10.1371/journal.pone.0247493)
Supplement: S1 Appendix — (DOCX) [file pone.0247493.s001.docx]

CREDIT ROSTER – DWYER PAPER – OFID 2018

**The INSIGHT FLU002 and FLU003 Study Group**

**International coordinating centers:**

**Copenhagen:** Bitten Aagaard, Álvaro H.  D. Borges, Alessandro CozziLepri, Marius Eid, Per O.  Jansson, Marianne Jeppesen, Zillah Maria Joensen, Ruth Kjærgaard Pedersen, Jens Lundgren, Birgit Riis Nielsen, Mary Pearson, Lars Peters, Tavs Qvist.

**London:** Brian Angus, Abdel Babiker, Rachel Bennett, Nafisah Braimah, Yolanda Collaco-Moraes, Adam Cursley, Fleur Hudson, Sarah Pett, Charlotte Russell, Helen Webb.

**Sydney:** Dianne Carey, David Courtney-Rodgers, Sean Emery, Pamela Shaw.

**Washington:** Fred Gordin, Adriana Sanchez, Barbara Standridge, Michael Vjecha.

**Statistical and Data Management Center, Minneapolis, Minnesota:** Kate Brekke, Megan Campbell, Eileen Denning, Alain DuChene, Nicole Engen, Michelle George, Merrie Harrison, James D. Neaton, Ray Nelson, Siu-Fun Quan, Terri Schultz, Deborah Wentworth.

**Specimen repositories and laboratories:** John Baxter, Shawn Brown (Leidos Biomedical Research, Inc.), Marie Hoover (ABML).

**National Institute of Allergy and Infectious Disease/Leidos:** John Beigel, Richard T.  Davey Jr., Robin Dewar, Erin Gover, Rose McConnell, Julia Metcalf, Ven Natarajan, Tauseef Rehman, Jocelyn Voell.

**Institute of Clinical Pathology and Medical Research, NSW Health Pathology, Westmead Hospital and University of Sydney, Westmead, New South Wales, Australia:** Dominic E. Dwyer, Jen Kok.

**Centers for Disease Control and Prevention, Atlanta, Georgia:** Timothy M. Uyeki.

**Community representative:** David Munroe.

**Clinical site investigators by country (n = number of participants enrolled):**

**Argentina** (n=1567): Damian Aguila, Maria Fernanda Alzogaray, Maria Fernanda Ballesteros, Laura Barcan, Laura Barcelona, Waldo Belloso, Veronica Berdiñas, Pablo Bonvehi, Juan Pablo Caeiro, Veronica Cisneros, Ana Crinejo, Daniel David, Luz Doldan, Juan Ebenrstejin, Flavio Lipari, Ana Lopardo, Gustavo Lopardo, Marcelo Losso, Pablo Lucchetti, Sergio Lupo, Laura Moreno Macias, Alejandra Moricz de Tesco, Analia Mykietiuk, Estaban Nannini, Gabriel Nieto, Laura Nieto, Luciana Peroni, Ignacio Retta, Patricia Rodriguez, Marisa Sanchez, Pablo Sanchez, Mariana de Paz Sierra, Silvina Tavella, Elena Temporiti, Liliana Trape, Ines Vieni, Eduardo Warley, Diego Yahni, Abel Humberto Zarate.

**Thailand** (n=895): Anchalee Avihingsanon, Kanlaya Charoentonpuban, Ploenchan Chetchotisakd, Peeraporn Kaewon, Naphassanant Laopraynak, Weerawat Manosuthi, Kanitta Pussadee, Opass Putcharoen, Kiat Ruxrungtham, Gompol Suwanpimonkul, Sasiwimol Ubolyam.

**United States** (n=654): Roberto Arduino, Barbara Atkinson, Taryn M. Aulicino, Jason V. Baker, Cindy Bardascino, Caitlin Bass, John D. Baxter, Mark Beilke, Beverly D. Bentley, Mary Lee Bertrand, Ann B. Brown, June Carbonneau, Richard Cindrich, Patty Coburn, Calvin J.  Cohen, Linda Clark, Shirley Cummins, Paul Dassow, Jack A. DeHovitz, Nila J. Dharan, Leslie Faber, Marti Farrough, Matthew Freiberg, Edward Gardner, Kimberly Jo Garrett, Christiane Geisler, Marshall Glesby, Julia Green, Joanne Grenade, Edie Gunderson, John Gunter, Kirsis Ham, Susan Holman, Valery Hughes, Christopher Hurt, Mary Johnson, Glory Koerbel, Susan Koletar, Audrey Lan, Rodger MacArthur, Cheryl Marcus, Norm Markowitz, Maria Laura Martinez, Karen McLaughlin, Raquel Nahra, Mary Jane Nettles, Daniel Nixon, Richard Novak, Kathleen Nuffer, Hannah B.  Olivet, Bola Omotosho, Armando P. Paez, Marta Paez-Quinde, Sonija Parker, Namrata Patil, Hari Polenakovik, Sandra Powell, Rachel A. Prosser, Nancy A. Reilly, Paul F. Riska, Stacey Rizza, Robert Schooley, Marla Schwarber, James Scott, Gary L. Simon, Jon Sivoravong, Daniel J. Skiest, Clemencia Solorzano, Rita Sondengam, Nicole Swanson, Ellen Tedaldi, Zelalem Temesgen, Doug Thomas, Bill Thron, Colleen Traverse, David E.  Uddin, Daniel Z.  Uslan, Marina Vasco, William M. Vaughan, Isabel Vecino, Barbara Wade, Catrice Walker, Kathy Watson, Vicky Watson, David Wohl, Cameron R. Wolfe.

**Belgium** (n=549): Leslie Andry, Mireille Bielen, Nathan Clumeck, Eric Florence, Kabamba Kabeya, Jolanthe Sagaer, Jozef Weckx.

**Greece** (n= 232): Olga Anagnostou, Anastasia Antoniadou, George Daikos, Vicky Gioukari, Ioannis Kalomenidis, Maria Kantzanou, Georgios Koratzanis, Nikolaos Koulouris, Efstratios Maltezos, Symeon Metallidis, Vlassis Polixronopoulos, Helen Sambatakou, Athanasios Skoutelis, Giota Touloumi, Nikolaos Vasilopoulos.

**Australia** (n=229): Mark Bloch, Nicky Cunningham, Dominic E. Dwyer, Sian Edwards, Julian Elliott, Jill Garlick, Philip Habel, Fiona Kilkenny, Helen Lau, Karen MacRae, John McBride, Richard Moore, Isabel Prone, Ristila Ram, Sue Richmond, Norm Roth, Tuck Meng Soo, Jo-Anne Thompson, Trina Vincent, Emanuel Vlakahis, Rachel Woolstencroft.

**United Kingdom** (n=199): David Chadwick, Tristan Clarke, Jane Democratis, David Dockrell, Robert Heyderman, Ben Jeffs, Stefan Kutter, Martin Llewelyn, Jane Minton, Melanie Newport, Ashley Price.

**Peru** (n=193): Carlos Benites, Raul Castillo, Romina Chinchay, Eva Cornelio, Maria Guevara, Luis Gutierrez, Jose Hidalgo, Alberto La Rosa, Yvett Pinedo, Maria Saenz, Juan Vega.

**Denmark** (n=180): Bente Baadegaard, Karen Bach, Philippa Collins, Jan Gerstoft, Lene Hergens, Lene Pors Jensen, Zillah Maria Joensen, Gitte Kronborg, Iben Rose Loftheim, Henrik Nielsen, Lars Oestergaard, Court Pedersen, Jens Aage Stauning, Svend Stenvang Pedersen, Yordanos Yehdego.

**Germany** (n=170): Frank Bergmann, Christoph Boesecke, Johannes R.  Bogner, Norbert Brockmeyer, Christine Czaja-Harder, Rika Draenert, Gerd Fätkenheuer, Hartwig Klinker, Tim Kümmerle, Clara Lehmann, Vera Müller, Andreas Plettenberg, Jürgen Rockstroh, Stefan Schlabe, Wolfgang E. Schmidt, Dirk Schürmann, Gundolf Schüttfort, Ulrich Seybold, Christoph Stephan, Albrecht Stoehr, Klaus Tillmann, Susanne Wiebecke, Timo Wolf.

**Spain** (n=163): Jose Arribas, Javier Carbone, Eduardo Fernández Cruz, David Dalmau, Vincente Estrada, Patricia Herrero, Hernando Knobel, Paco López, Rocío Montejano, José Sans Moreno, José Ramón Paño, Begoña Portas, Maria Rodrigo, Pilar Romero, Domingo Sánchez-Sendín, Vincente Soriano.

**Poland** (n=130): Elzbieta Bakowska, Andrzej Jerzy Horban, Brygida Knysz, Karolina Pyziak Kowalska, Anna Zubkiewicz-Zarebska.

**Estonia** (n=99): Kerstin Kase, Helen Mülle, Kai Zilmer.

**Chile** (n=24): Gladys Allendes, Jimena Flores, Rebeka Northland, Carlos Perez, Isabel Velasco, Marcelo Wolff.

**China** (n=24): Man-Yee Chu, Tak-chiu Wu.

**Austria** (n=20): Heinz Burgmann, Selma Tobudic.

**Japan** (n=14): Mayumi Imahashi, Junji Imamura, Yasumasa Iwatani, Ayumi Kogure, Masashi Nakahata, Wataru Sugiura, Yoshiyuki Yokomaku.

**Norway** (n=8): Anne Maagaard.

Figure 1S: Distribution of Mortality Outcome by Kynurenine and Tryptophan Tertiles


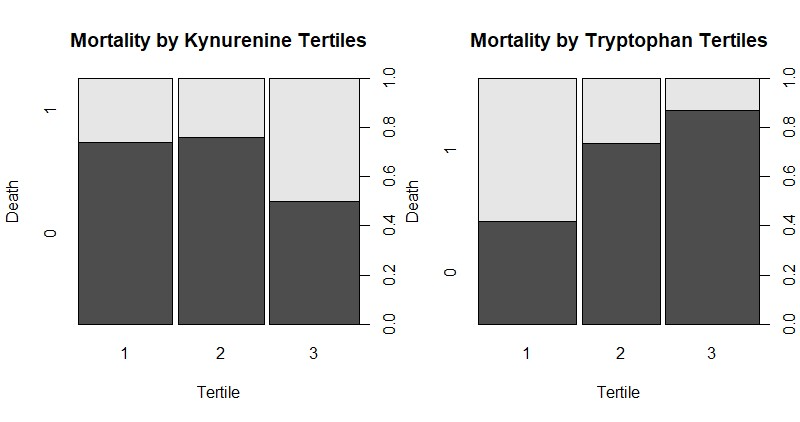


Table 1S: Number of Metabolites in the Original Data Set vs. Number of Metabolites Remaining after removing Metabolites with at Least 10% of Values below the LOD for Cases and Controls per Metabolite Family

|  | Number of Metabolites | | | Percent Reduction |
| --- | --- | --- | --- | --- |
|  | Original | Included in Analysis | Removed |  |
| Acylcarnitines | 55 | 16 | 39 | 70.91% |
| Amino Acids | 21 | 21 | 0 | - |
| Biogenic Amines | 21 | 6 | 15 | 71.43% |
| Cholesterol Esters | 14 | 9 | 5 | 35.71% |
| Glycerides | 60 | 42 | 18 | 30.00% |
| Glycerophospholipids | 196 | 64 | 132 | 67.35% |
| Sphingolipids | 40 | 30 | 10 | 25.00% |
| Monosaccharide | 1 | 0 | 1 | 100.00% |
